# Cell Therapy Using Bone Marrow-Derived Stem Cell Overexpressing BMP-7 for Degenerative Discs in a Rat Tail Disc Model

**DOI:** 10.3390/ijms17020147

**Published:** 2016-01-22

**Authors:** Jen-Chung Liao

**Affiliations:** Department of Orthopedics Surgery, Bone and Joint Research Center, Chang Gung Memorial Hospital, Chang Gung University, No._5, Fu-Shin Street; Kweishian, Taoyuan 333, Taiwan; jcl1265@adm.cgmh.org.tw; Tel.: +886-3-328-1200 (ext. 2423); Fax: +886-3-327-8113

**Keywords:** bone marrow stem cell, bone morphogenetic protein 7, baculovirus, disc degeneration, rat tail, needle puncture

## Abstract

Degenerative discs can cause low back pain. Cell-based transplantation or growth factors therapy have been suggested as a strategy to stimulate disc regeneration. Bone marrow-derived mesenchymal stem cells (BMDMSC) containing bone morphogenetic protein-7 (BMP-7) gene were constructed. We evaluated the effectiveness of these BMP-7 overexpressing cells on degenerative discs in rat tails. *In vitro* and *in vivo* studies were designed. In the first stage, the rats were divided into two group according to discs punctured by different needle gauges (18 gauge and 22 gauge). In the second stage, the ideal size of needle was used to induce rat tail disc degeneration. These animals are divided into three groups according to timing of treatment (zero-week, two-week, four-week). Each group was divided into three treating subgroups: control group, BMDMSC group, and Baculo-BMP-7-BMDMSC group. Each rat undergoes radiography examination every two weeks. After eight weeks, the discs were histologically examined with hematoxylin and eosin stain and Alcian blue stain. The 18-gauge group exhibited significant decrease in disc height index (%) than 22-gauge group at eight weeks at both Co6-7 (58.1% ± 2.8% *vs.* 63.7% ± 1.0%, *p* = 0.020) and Co8-9 discs (62.7% ± 2.8% *vs.* 62.8% ± 1.5%, *p* = 0.010). Baculo-BMP-7-BMDMSCs group showed significant difference in disc height index compared to the BMDMSCs group at both Co6-7 (93.7% ± 1.5% *vs.* 84.8% ± 1.0%, *p* = 0.011) and Co8-9 (86.0% ± 2.1% *vs.* 81.8% ± 1.7%, *p* = 0.012). In Baculo-BMP-7-BMDMSCs group, the zero-week treatment subgroup showed significant better in disc height index compared to two-week treatment group (*p* = 0.044), and four-week treatment group (*p* = 0.011). The zero-week treatment subgroup in Baculo-BMP-7-BMDMSCs group also had significant lower histology score than two-week treatment (4.3 *vs.* 5.7, *p* = 0.045) and four-week treatment (4.3 *vs.* 6.0, *p* = 0.031). In conclusion, Baculo-BMP-7-BMDMSC can slow down the progression of disc degeneration, but could not provide evidence of regeneration. Early treatment might obtain more distinct results.

## 1. Introduction

Degenerative disc disease is one of the most common causes of low back pain. After failure of conservative treatment, the fusion procedure remains the “gold standard” treatment for this problem. Such a fusion procedure predisposes the juxtafusion segments and accelerates the adjacent segment degeneration [1,2,3]. As a result, the concept of “non-fusion” has become popular in recent years [4,5]. Many new treatment modalities and devices were developed, but the results were still not convincing. These problems included wear debris, loosening of fixation, spontaneous fusion, and carrying high risks in revision surgeries [6,7,8]. Advances in molecular biology have enabled a more biological approach to degenerative discs. The regeneration of the degenerated disc with growth factors, cell-based therapy is being pursed [9,10,11]. Among these growth factors, bone morphgenetic protein-7 (BMP-7) has shown to stimulate the metabolism of disc cells *in vitro* [12], the ability of growth factors therapies to stimulate degenerative disc repair *in vivo* has not been proven [13]. Without a good carrier, the external growth factor is easy to leak out and does not endure a few hours inside the disc. Cell transplantation using a mesenchymal stem cell type has been suggested as a potential strategy [14,15]: bone marrow derived mesenchymal stem cells (BMDMSC) can differentiate into chondroblasts and assist endogenous cell population inside the disc. If the transplanted cells can assist the endogenous cell population and carry therapeutic gene that could be delivered *in vivo* on a prolonged basis, it has a greater chance to stop the degenerative process or achieve a successful repair. Since 1995, baculovirus has been employed to deliver genes into numerous mammalian cells, including neural cells, fibroblasts, human chondrocytes, pancreatic islet cells, and human bone marrow stem cells [16,17,18,19,20]. Additionally, the baculovirus entry into mammalian cells does not result in a visible cytopathic effect [21]. Due to these advantages, the baculovirus is thought to be an ideal vector in gene therapy.

The rat-tail has been found to serve as an appropriate model because their intervertebral discs are easily accessible and are of an adequate size to study disc surgery [22]. The purpose of this study is to establish a disc degeneration model by puncturing rat-tail discs with different sizes of needle. Radiography and histology are performed to monitor the progress of disc degeneration. Furthermore, we also want to evaluate the effectiveness of genetically-modified mesenchymal stem cells overexpressing BMP-7 on the degenerative disc. We hypothesize that these cells can survive within the disc, overexpress target genes, and enhance disc regeneration.

## 2. Results

### 2.1. The Effect of Different Needle Sizes

#### 2.1.1. Radiography Results

To find out which needle size induces more severe degeneration in discs, we used 18- and 22-guage needles to puncture the coccygeal discs of rat tails. No significant difference was observed in the control discs over an eight-week period. Both 18-gauge and 22-gauge groups caused a gradual decrease in changes of disc height index (%DHI). However, in Co6-7, the 18-gauge group exhibited a significant decrease in %DHI than the 22-gauge group at eight weeks (58.1% ± 2.8% *vs.* 63.7% ± 1.0%, *p* = 0.020). Similarly, in Co8-9, a significant decrease in %DHI was found in the discs punctured with 18-guage needle compared with those with 22-guage eight weeks after puncture (62.7% ± 2.8% *vs.* 69.8% ± 1.5%, *p* = 0.010). The finding suggested that an 18-gauge puncture caused more significant damage to the discs than a 22-gauge puncture.

#### 2.1.2. Histology Results

**Figure 1:**
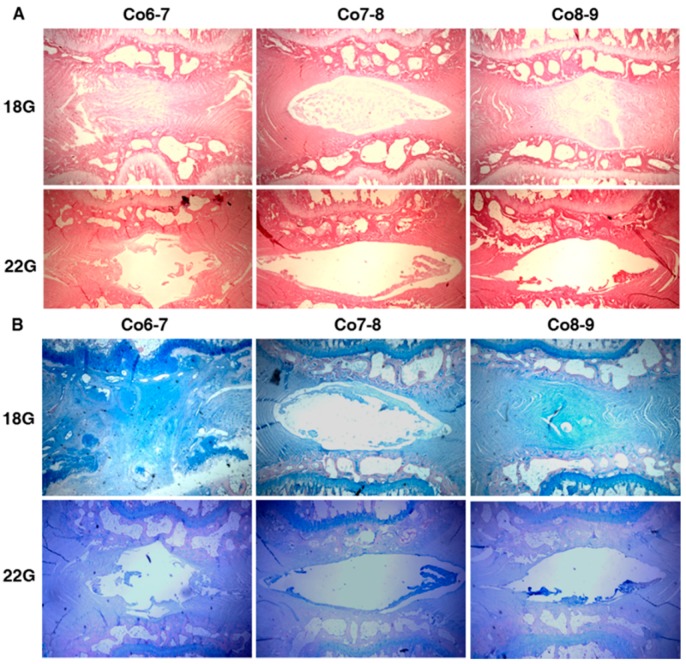
(**A**) Hematoxylin and eosin stain and (**B**) Alcian blue stain for comparing the morphological and proteoglycan changes of control and punctured discs with 18-guage and 22-guage needles (original magnification: 40×). The control disc, Co7-8, showed a rounded nucleus pulposus and a well-organized annulus fibrosus with a well-defined border. The punctured discs, Co6-7 and Co8-9, showed a clustered NP and a disordered AF with an interrupted border.

Comparing to the control discs, the architectural disorder in the annulus fibrosus (AF) and nucleus pulposus (NP) was observed. The control discs showed a rounded NP and a well-organized AF with a well-defined border between them, whereas the punctured discs showed a clustered NP and a disordered AF with an interrupted border. There were no significant differences in histological scores for both stains in the control discs. The histological score of Hematoxylin and Eosin (H and E) staining between 18-gauge (G) and 22-gauge (G) groups showed significant difference for Co6-7 (5.3 ± 0.4 *vs.* 3.8 ± 0.4, *p* = 0.003), and Co8-9 (5.0 ± 0.3 *vs.* 3.5 ± 0.4, *p* = 0.012) (Figure 1A) .The histological score of Alcian blue stain between 18-gauge and 22-gauge groups also showed significant difference for Co6-7 (4.5 ± 0.5 *vs.* 3.0 ± 0.1, *p* = 0.001), and Co8-9 (5.0 ± 0.4 *vs.* 3.5 ± 0.3, *p* = 0.015) (Figure 1B). The disc punctured by an 18 G needle obtained a more degenerative change than that punctured by a 22 G needle at final follow up, so we chose an 18 G needle to induce disc degeneration in the later study.

### 2.2. In Vitro Study: ELISA for BMP-7 Production

One day after transduction, ELISA demonstrated that baculoviral BMP-7 transduced mesenchymal stem cells produced mean BMP-7 levels of 289 pg/mL. The baculoviral BMP-7 transduced mesenchymal stem cells exhibited near five-fold greater BMP-7 production than untransduced bone marrow stem cells (*p* < 0.001).

### 2.3. The Effect of Baculoviral BMP-7 Transduced Mesenchymal Stem Cells on Punctured Discs

Virus titration by 50% tissue culture infective dose (TCID50) was converted to plaque-forming units (pfu), and resulted in 1.35 × 10^7^ pfu/mL. Bone marrow-derived mesenchymal stem cells were infected with virus at a multiplicity of infection (MOI) of 25 pfu/cell. 1 × 10^4^ BMDMSCs or Baculo-BMP-7-BMDMSCs were injected into each disc.

#### 2.3.1. Comparisons between Two Different Injected Cells at Different Time-Points of Treatments

**Figure 2:**
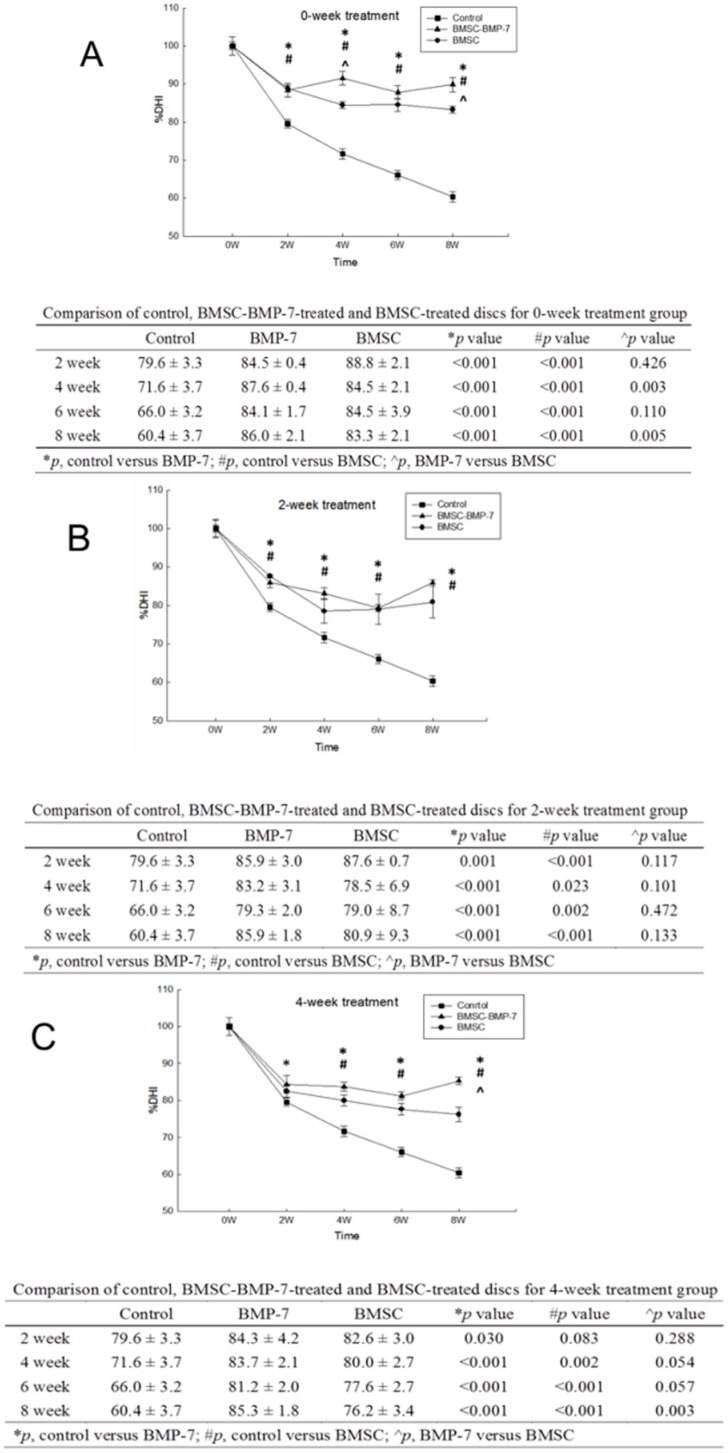
(**A**) Zero-week treatment group. The ratio disc height change at different methods (control, BMDMSCs, Baculo-BMP-7-BMDMSCs) and time points. The Baculo-BMP-7-BMDMSC could maintain better disc height; (**B**) two-week treatment group. Comparing to the control subgroup, BMMSCs and , Baculo-BMP-7-BMDMSCs could sustain more disc height; (**C**) four-week treatment group. There was still a trend that the Baculo-BMP-7-BMDMSCs subgroup had more ability to slow down disc-narrowing induced by disc puncture.

The Baculo-BMP-7-BMDMSCs group showed significant difference in %DHI compared to the BMDMSCs group at both Co6-7 (93.7% ± 1.5% *vs.* 84.8% ± 1.0%, *p* = 0.011) and Co8-9 (86.0% ± 2.1% *vs.* 81.8% ± 1.7%, *p* = 0.012) (Figure 2A). For two-week treatment, injecting the cells two weeks after the puncture, there were no significant differences between the BMDMSCs and Baculo-BMP-7-BMDMSCs groups for both Co6-7 (84.7% ± 0.5% *vs.* 81.3% ±10.7%, *p* = 0.343) and Co8-9 (87.1% ± 1.9% *vs.* 80.5% ± 7.7%, *p* = 0.143) (Figure 2B). For four-week treatment, injecting the cells four weeks after the puncture, the Baculo-BMP-7-BMDMSCs group showed significant difference in %DHI compared to the BMDMSCs group at both Co6-7 (84.0% ± 1.1% *vs.* 72.8% ± 0.8%, *p* = 0.020) and Co8-9 (86.6% ± 1.7% *vs.* 79.5% ± 0.8%, *p* = 0.040) (Figure 2C).

#### 2.3.2. Comparisons inside Three Different Time-Points of Treatment with Two Different Injected Cells

For injecting Baculo-BMP-7-BMDMSCs to Co6-7, the zero-week treatment group showed to be significantly better in %DHI compared to two-week treatment group (93.7% ± 1.5% *vs.* 84.7% ± 0.5%, *p* = 0.044), and four-week treatment group (93.7% ± 1.5% *vs.* 84.0% ± 1.1%, *p* = 0.011). For injecting Baculo-BMP-7-BMDMSCs to Co8-9, there were no significant difference when comparing the %DHI between zero-week and two-week treatment (*p* = 0.279), and zero-week and four-week treatment (*p* = 0.393).

For injecting only BMDMSCs to Co6-7, the zero-week treatment group showed to be significantly better in %DHI compared to four-week treatment group (84.8% ± 1.0% *vs.* 72.2% ± 0.8%, *p* = 0.042), whereas no significance was observed between the zero-week and two-week treatment (84.8% ± 1.0% *vs.* 81.3% ± 10.7%, *p* = 0.345). For injecting only BMDMSCs to Co8-9, there were no significant difference when comparing the %DHI between zero-week and two-week treatment (*p* = 0.418), and zero-week and four-week treatment (*p* = 0.194).

**Figure 3:**
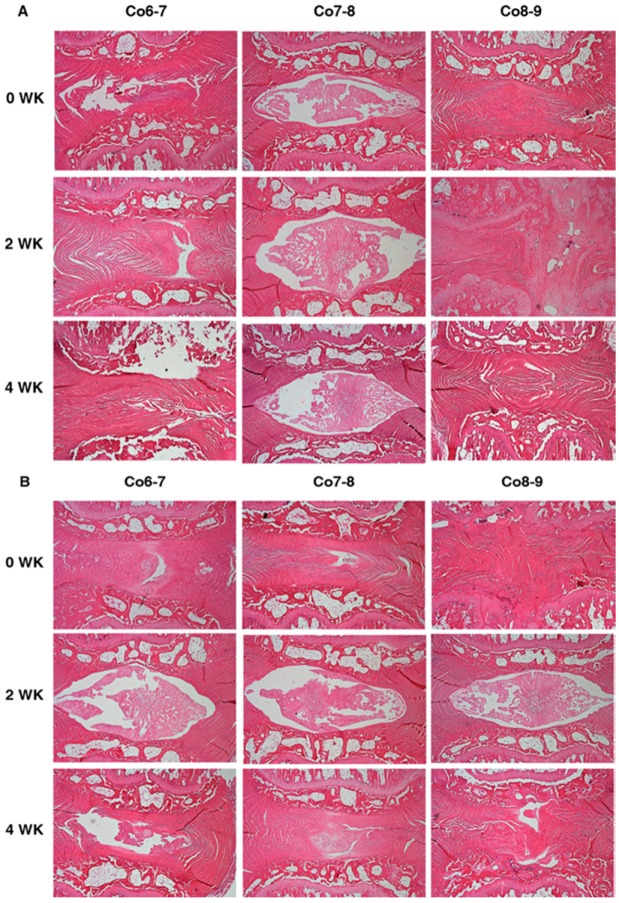
Hematoxylin and eosin staining for comparing the morphological change of injecting (original magnification 40×) (**A**) Baculo-BMP-7-BMDMSCs and (**B**) BMDMSCs only at different treatment time-points (zero, two, and four weeks after puncture). Co7-8 is the control disc with no injection. Co6-7 and Co8-9 are the punctured discs with injection of cells.

**Figure 4:**
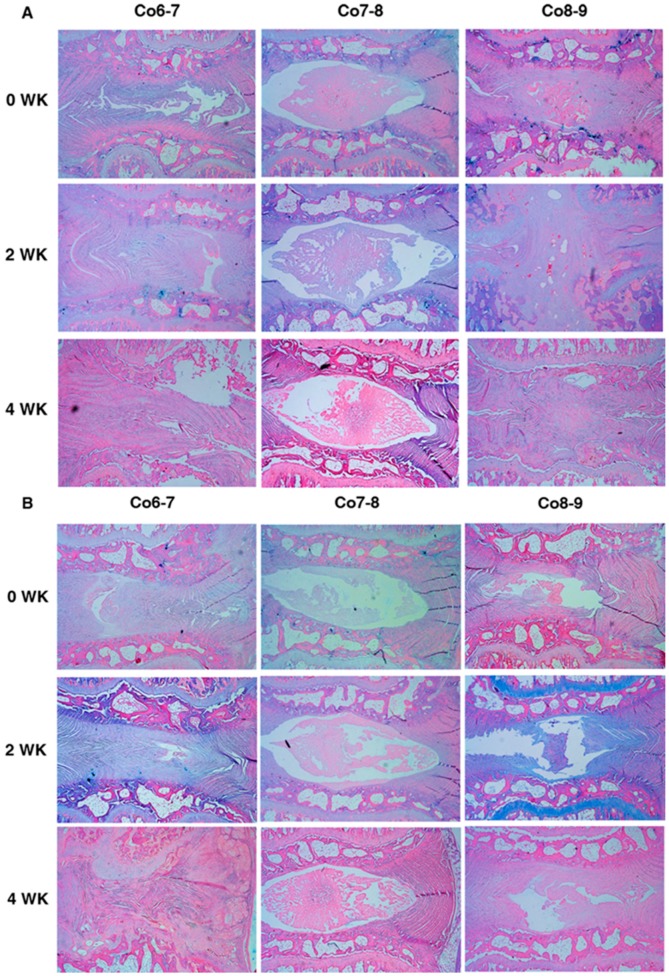
Alcian blue stain for comparing the proteoglycan change of injecting (original magnification: 40×). (**A**) Baculo-BMP-7-BMDMSCs and (**B**) BMDMSCs only at different treatment time-points (zero, two, and four weeks after puncture). Co7-8 is the control disc with no injection. Co6-7 and Co8-9 are the punctured discs with injection of cells.

As analyzing the effect of baculoviral BMP-7 with different treating time-points on histology results; at week 0, the Baculo-BMP-7-BMDMSCs group demonstrated significant better scores than the BMDMSCs group at Co6-7 (4.3 ± 0.5 *vs.* 5.8 ± 0.4, *p* = 0.008), and Co8-9 (4.3 ± 0.6 *vs.* 5.6 ± 0.5, *p* = 0.047). There were no significant differences between these two groups at week 2 and 4. As analyzing the effect of treatment time with different cells, the histological score of H & E staining revealed that the zero-week treatment group had significant lower score than two-week treatment (4.3 ± 0.5 *vs.* 5.7 ± 0.5, *p* = 0.045) and four-week treatment (4.3 ± 0.5 *vs.* 6.0 ± 0.3, *p* = 0.031) for injecting Baculo-BMP-7-BMDMSCs to Co6-7 and Co8-9 (Figure 3A). However, there was no significant difference between different treatment times for injecting BMDMSCs only to Co6-7 and Co8-9 (Figure 3B). Furthermore, the histological score of Alcian blue stain showed no significant difference for Co6-7 and Co8-9 in all comparisons (Figure 4A,B).

## 3. Discussion

Needle puncture at the intervertebral disc has become a popular method to create an animal model that mimics human disc degeneration. Rodent, rabbit, canine, pig, and sheep were used for the puncture model [23,24,25,26,27,28]. Although the higher species have more cellular components similar to humans, the high cost and difficult availability are their limitations. Needle puncture at the rodent tail disc has established by Han *et al.* [29] in 2008. The advantages of this method are simple, inexpensive, high reproducibility. The rate of degeneration in rat tai l disc puncture model is related the depth of puncture. Keorochana *et al.* [30] also performed a needle puncture model in rat tail disc, and evaluated by radiography, MRI, and immunohistochemistry method. The results showed larger needle gauges produced more degenerative discs. In our study, we used 18 G and 22 G needles to puncture rat tail discs. The 18 G group demonstrated narrower disc height and higher histology scores (more degenerative) at the eight-week time point than that of the 22 G group. To evaluate the efficacy of BMP-7 producing bone marrow stem cells on the degenerative disc, we choose the 18 G needle punctured disc in the second stage study because 18 G needle can reproduce a more degenerative disc for later comparisons. Similarly, Elliott *et al.* [31] used 27 G and 33 G needles to puncture rat tail discs; their data showed a larger size of needle could induce greater injury because the size of a 27 G needle occupied 52% of the disc height of a rat tail disc. These damaged rat tail discs were degenerated initially via annulus fibrosus tear with nucleus pulposus leakage. With various torsion or compression stress on the injured annulus fibrosus leads to a further degeneration [32,33].

Growth factors such as BMP-2 or BMP-7 have been used to induce spinal fusion for years. FDA indications in human are lumbar interbody fusion for BMP-2 and lumbar posterolateral fusion for BMP-7. The literatures related to the role of growth factors on the intervertebral disc are not abundant. BMP-7, a member of the transforming growth factor-B superfamily, has been demonstrated that *in vitro* stimulation of intervertebral disc cells can promote synthesis and organization of proteoglycan of extracellular matrix [34,35]. An *et al.* [36] has conducted an *in vivo* study in rabbit normal intervetebral discs that BMP-7 showed an ability to increase disc height and the peroteoglycan content of the nuclear pulposus at two weeks after injections; the collagen content of the annulus fibrosus was also significant increase at four weeks after injections. The healthy disc cells inside the nucleus pulposus can be stimulated by BMP-7 to increase production of proteoglycan content; these increased proteoglycan molecules act to attract water, therefore, the intervertebral disc height of the normal disc increases after BMP-7 injection [37].

All content and disc cells are decreased in a degenerative disc, therefore, pure growth factor is not suitable for a degenrative disc because no adequate disc cells exist for reaction. Mesenchymal stem cells isolated from bone marrow aspiration provide an unlimited cell source high proliferation activity and the potential to differentiate into several cell lineages, including chondrocyte-like cells and disc-like cells [38]. Risbud *et al.* [39] conducted an *in vitro* study that bone marrow-derived mesechymal stem cells were cultured in medium with transforming growth factor-B1 under hypoxia (2% O_2_) condition, these mesenchymal stem cells can differntiate into a phenotype consistent with that of the nucleus pulposus. Furthermore, mesenchymal stem cells also have ability to up-regulate the viability of nucleus pulposus cells when both cells are cultured in a certain co-culture system [40,41]. These findings imply that mesenchymal stem cells can be used to regenerate a degenerative disc. In a rabbit model, transplantation of mesencymal stem cells in the degenerative disc can maintain 91% disc height valus and 81% MRI intensity of the normal control group; but the sham-operated group obtained only 67% disc height values and 60% MRI intensity. In addition, restoration of the proteoglycan is also higher in the mesenchymal stem cells transplantaion group, so the authors concluded that the transplantation of mesenchymal stem cells can provide an ability to regenerate the degenerative disc [14]. Similar results were also seen in the canine model; the authors also indicated that the degenerative intervertebral discs are a tissue with immune privilege, which make the transplanted mesenchymal stem cells survive and differentiate into a certain cells to regenerate or stop ongoing degeneration in the degenerative disc [42].

Virus-mediated delivery of growth factors have been developed successfully in inducing healing in a posterolateral spinal fusion model or a long bone defect model [43,44,45]. By this method, the transfected cells can secrete growth factors to stimulate bone repair with an autocrine and paracrine activities. In the present model, we used BMDMSCs as the protein delivery system. MSCs have gained considerable interest because their self-renewing ability and multi-potential. Theoretically, the transduced MSCs are inherently transformed into chondrocytes or disc-like cells. The over-expressed BMP-7 production by gene modified BMDMSCs might stimulate the remaining disc cells inside the degenerative disc to act normally. On the contrary, the secreted BMP-7 also could stimulate BMDMSCs to transform into disc-like cells, to maintain the disc content, to prevent further disc degeneration. Furthermore, BMDMSCs would interact with the remaining disc cells into a positive way. In the present study, the Baculo-BMP-7-BMDMSCs could secrete more BMP-7 than the BMDMSCs in the *in vitro* study. This implied the Baculo-BMP-7-BMDMSCs can over-express BMP-7. According to the studies of Griffith *et al.* [46] and Bellgrau *et al.* [47], the intervertebral disc was confirmed as a tissue with immune privilege. Hiyama *et al.* [41] demonstrated that the transplanted mesenchymal stem cells inside the intervertebral disc could express Fas-ligand, which is only found in tissue with immune privilege. One weak point of this study was that we did not provide evidence which demonstrated these transplanted cells survived and had function inside the degenerative disc. We did believe that these transplanted Baculo-BMP-7-BMDMSCs and BMDMSCs had functions in the degenerative disc because these treatment groups can maintain better disc heights and histology scores.

From the time effect to changes of the treatment, most of significant differences occurred between zero- and four-week time points of treatment between Baculo-BMP-7-BMDMSCs and BMDMSCs groups. There was no significant difference at the two-week time point of treatment between these two groups. From above results, the disc becomes degenerative rapidly during the first two weeks after disc puncture. No matter what kind of treatment, the degenerated disc could not be regenerated. Early intervention for a degenerative disc is a reasonable strategy to slow progression of disc degeneration.

## 4. Experimental Section

A total of 72 male Lewis rats at eight weeks of age were obtained from the Laboratory Animal Center, and this animal study was approved by the Institutional Animal care and Use Committee of Change Gung Memorial Hospital. These rats were divided into two groups. First, the rat-tail disc degeneration model was established by percutaneous needle puncture; second, the effectiveness of genetically-modified mesenchymal stem cells overexpressing BMP-7 on the degenerative discs was evaluated.

### 4.1. Establishment of Rat-Tail Disc Degeneration Model

**Figure 5:**
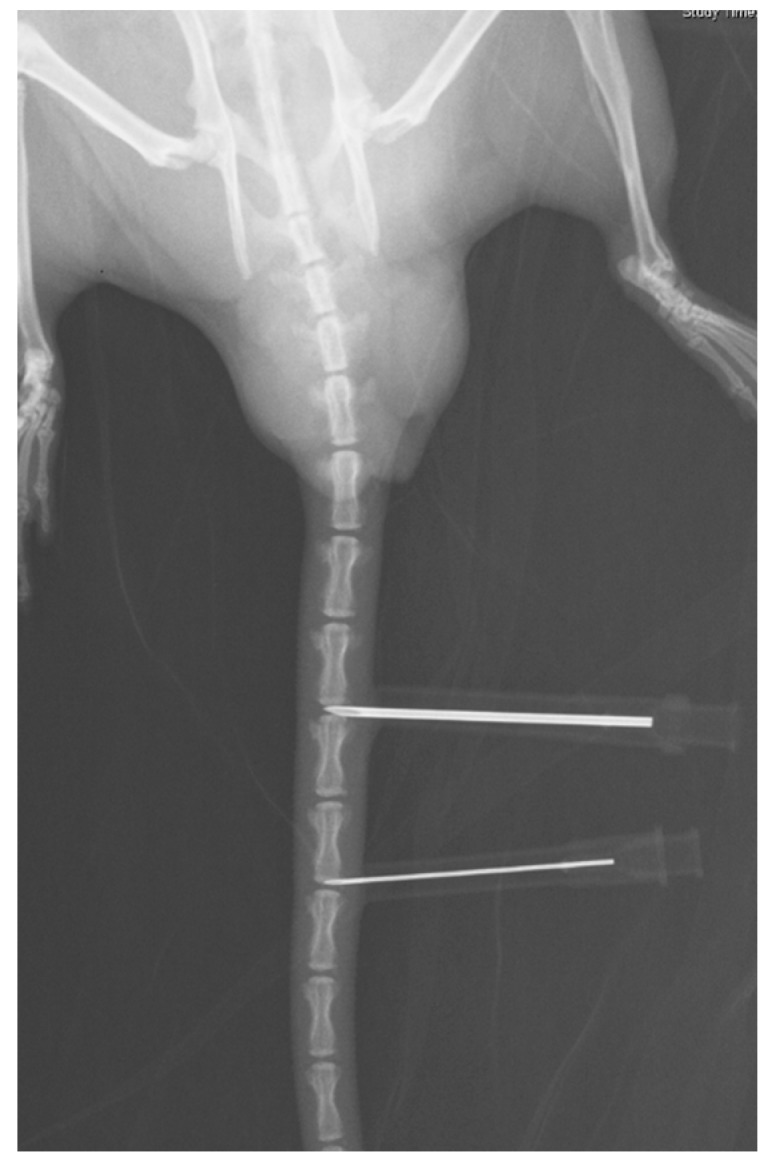
A radiograph demonstrating two needles puncturing Co6-7 and Co8-9 discs.

The rats are anesthetized by 1% isoflurane inhalation in a chamber. The rat is put in a prone position. Under the help of fluoroscope, the Co6-7 and Co8-9 discs are punctured with various size of needles (18, 22 gauge) (Figure 5). The Co7-8 intervertebral disc is left to be uninjured control. The needle is inserted at the center of the disc through the annulus fibrosus (AF) into the nucleus pulposus (NP) and held for 30 s. After removal of the needle, the wound is covered with gauze.

### 4.2. Radiography Analysis

At zero, two, four, six, and eight weeks post-surgery, rats from each group undergo radiography evaluation. The rat is placed prone. The tail is laid straight that create the vertical beam to the tail and focus at the target levels. Two independent observers who are not involved in the index operation performed the radiographic assessment. The data of disc height was expressed as disc height index (DHI) and was calculated by averaging the measurements of disc height obtained from one fourth, middle, and three-fourths portion of adjacent cranial endplate width and dividing that by the average of adjacent vertebral body heights at the same point. This method has been published by Han *et al.* [29].

### 4.3. Histology Analysis

After euthanasia, the whole discs with adjacent vertebrae is dissected, and the specimens fixed in 10% formalin, decalcified using 10% decalcifying solution hydrochloride acid, washed with running tap water, and then transferred to 75% ethanol. The specimens are embedded in paraffin blocks. The tissue blocks are sectioned at 5 μm, and stained with hematoxylin and eosin (H and E) and Alcian blue stain. The slides are evaluated by two independent observers and graded using a histologic grading scale based on the histologic appearance of the NP, AF, and proteoglycan (PG) staining characteristics. H and E stain mainly determines the cellular and morphological changes in both NP and AF. The scores range from 0 to 3, where normal is the zero point for each portion (NP and AF) [48]. A total of six points show the most severe degeneration. The Alcian blue stain was used to evaluate the PG change within the disc. The range of grading is from normal (Grade 0) to severe loss of staining (Grade 4), according to Norcross *et al.* [49].

### 4.4. Rat Bone Marrow Cell Harvest, Culture, and Gene Transfer

#### 4.4.1. Isolation of Rat Bone Marrow Cells, Rat Bone Marrow Stem Cell Cultures

Bone marrow cells are harvested from the tibia and femur of four-week old female Lewis rats. The bone marrow cavities of the rats are flushed with Iscove’s Modified Dulbecco’s Medium (IMDM). The resulting cell suspension is centrifuged. The cell layer is plated and cultured in IMDM, supplement with glutamine and 10% heat-inactivated fetal bovine serum (FBS) at 5% CO_2_ and 37 °C. The culture medium is replaced twice a week along with the removal of the non-adherent cells. When confluent, the adherent cells are trypsinized and passaged at a 1:3 split. Passage 3 cells are used for transduction with baculovirus.

#### 4.4.2. Construction and Production of Baculoviral Vector

The recombinant baculovirus expressing BMP-7 under the cytomegalovirus immediate early (CMV-IE) promoter is constructed and designed as Bac-BMP-7. In brief, the CMV-IE promoter is polymerase chain reaction (PCR)-amplified from pcDNA3.1 (Invitrogen, Carlsbad, CA, USA) and suclones into pFastBac DUAL vector (Invitrogen). The human BMP-7 gene are amplified and cloned downstream of the CMV-IE promoter. The plasmid harboring BMP-7 gene is employed to construct the recombinant baculoviruses using Bac-To-Bac system (Invitrogen) according to the manufacturer’s instructions. The virus is further propagated in Spodoptera frugiperda (Sf-9) cell grown in suspension (200 mL) TNM-FH medium supplement with 10% FBS. The virus particles are collected three days after transfection and filtered through a 0.22 μM pore filter and concentrated at 80,000× *g* for 2 h at 4 °C. Viral titers are determined by end-point dilution method.

#### 4.4.3. Bone Marrow Stem Cell Transduction with Baculoviral Vectors

The bone marrow stem cells are seeded onto six-well plates (1 × 10^6^ cells/well) and cultured overnight. Prior to transduction, the culture medium is aspirated and the cells are washed once with PBS. At the multiplicity of infection (MOI) 25, a certain volume of virus supernatant is diluted to 200 μL (per well) with TNM-FH medium and then mixed with 800 μL PBS. After incubation for 24 h, the transduction medium is replaced with fresh IMDM for bone marrow stem cells.

#### 4.4.4. *In Vitro* BMP-7 Production Quantified by the Enzyme-Linked Immunosorbent Assay (ELISA)

The *in vitro* BMP-7 production by bone marrow cells, transduced bone marrow stem, or transduced bone marrow stem cells with baculo-BMP-7 is quantified in culture medium by an ELISA (R and D Systems, Minneapolis, MN, USA), according to the manufacture’s instruction. Before implantation in animals, a 24-h incubation culture medium is collected to analyze BMP-7 production. This test is to determine that whether Baculo-BMP-7-bone marrow stem cells could overexpress BMP-7 gene function.

#### 4.4.5. Different Treatment Modalities for Degenerative Discs

According to the results of the Step 1 study, we chose the ideal size of needle to induce Co6-7, Co8-9 coccygeal intervertebral disc degeneration (*n* = 60). These animals are divided into three groups according to timing of treatment (zero-week, two-week, four-week). Each group has 20 experiment rats and divides into four treating subgroup: BMDMSC (*n* = 10), and Baculo-BMP-7-BMDMSC (*n* = 10). 1 × 10^4^ BMDMSC or Baculo-BMP-7-BMDMSC cells were injected into the punctured disc.

Each rat undergoes radiography examination every two weeks. At eight weeks, all rats are sacrificed. The experiment discs are harvested. The discs of rats are histologically examined with H and E stain and Alcian blue stain.

## 5. Conclusions

Larger needle gauges induced more deterioration of rat tail discs. Baculo-BMP-7-BMDMSC can slow down the progression of disc degeneration, but could not provide evidence of regeneration. Early treatment might obtain more distinct results.
